# Polymer-Mediated Signal Amplification Mechanisms for Bioelectronic Detection: Recent Advances and Future Perspectives

**DOI:** 10.3390/bios15120808

**Published:** 2025-12-11

**Authors:** Ying Sun, Dan Gao

**Affiliations:** 1 College of Food Science, South China Agricultural University, Guangzhou 510642, China; yingsun@stu.scau.edu.cn; 2 Institute of Biopharmaceutical and Health Engineering, Tsinghua Shenzhen International Graduate School, Tsinghua University, Shenzhen 518055, China; 3 The State Key Laboratory of Chemical Oncogenomics, Key Laboratory of Chemical Biology, Tsinghua Shenzhen International Graduate School, Tsinghua University, Shenzhen 518055, China

**Keywords:** polymer materials, signal amplification, molecular recognition, bioelectronic sensing, conductive polymers

## Abstract

In recent years, polymer-mediated signal amplification has drawn wide attention in bioelectronic sensing. With the rapid progress of biosensing and flexible electronics, polymers with excellent electron–ion transport properties, tunable molecular structures, and good biocompatibility have become essential materials for enhancing detection sensitivity and interfacial stability. However, current sensing systems still face challenges such as signal attenuation, surface fouling, and multi-component interference in complex biological environments, limiting their use in medical diagnosis and environmental monitoring. This review summarizes the progress of conductive polymers, molecularly imprinted polymers, hydrogels, and composite polymers in medical diagnosis, food safety, and environmental monitoring, focusing on their signal amplification mechanisms and structural optimization strategies in electronic transport regulation, molecular recognition enhancement, and antifouling interface design. Overall, polymers improve detection performance through interfacial electronic reconstruction and multidimensional synergistic amplification, offering new ideas for developing highly sensitive, stable, and intelligent biosensors. In the future, polymer-based amplification systems are expected to expand in multi-parameter integrated detection, long-term wearable monitoring, and in situ analysis of complex samples, providing new approaches to precision medicine and sustainable environmental health monitoring.

## 1. Introduction

Bioelectronic detection, as a representative interdisciplinary field, has exhibited significant potential in medical diagnostics, food safety, and environmental monitoring in recent years [1,2,3]. Typically, such detection methods integrate highly selective biorecognition elements, including antibodies, nucleic acid aptamers, enzymes, and molecularly imprinted polymers (MIPs), with signal transduction modules such as electrochemical, optical, or impedance-based systems. By capturing target molecules and converting the resulting subtle physical or chemical variations into measurable signals, these systems enable rapid, sensitive, and specific detection [4,5]. However, as detection targets extend to low-abundance biomarkers and complex environmental samples, the original signals often become extremely weak and susceptible to interference from nonspecific background components, leading to reduced sensitivity, specificity, and system stability [6]. Against this disadvantage, the development of effective signal amplification strategies has emerged as a key challenge for advancing the performance and practical applications of bioelectronic detection technologies.

Current signal amplification strategies primarily include nanomaterial-based enhancement, electrical property modulation, strand-displacement reactions, and enzyme-assisted amplification. In nanomaterial-based systems, direct contact between nanomaterials and biorecognition elements interacting with the target analyte can significantly strengthen the output signal [2,7,8,9,10]. Although these approaches have improved detection sensitivity to a certain extent, they still suffer from inherent limitations such as poor stability, strong environmental dependence, and insufficient integration with device systems. In contrast, polymer-based materials, featuring programmable molecular structures, modifiable multifunctional sites, and multimodal tunability in electrical, optical, and chemical responses, can not only enhance system stability and environmental adaptability but also precisely regulate molecular recognition and signal transduction at the molecular level. This enables them to overcome the shortcomings of traditional strategies in terms of stability, controllability, and device compatibility [11,12,13,14]. Furthermore, the high compatibility of polymer materials with electronic devices provides new opportunities for the development of highly sensitive, integrable, and intelligent detection platforms [15,16].

In recent years, the rapid advancement of polymer materials in fields such as materials chemistry, nanotechnology, and biomedicine has provided a solid foundation for their application in bioelectronic detection [17,18]. Various types of polymers, including conductive polymers (CPs), hydrogels, polymer nanoparticles, and MIPs, have been employed to construct signal amplification systems. These materials enhance electronic or optical responses, improve the stability of recognition elements, and optimize interfacial interactions with detection devices, thereby enabling efficient detection of low-abundance analytes. This demonstrates that polymer materials can be flexibly integrated into diverse detection platforms, such as electrochemical, optical, and impedance-based systems, to effectively enhance sensitivity, specificity, and reliability. In this review, a summary of recent advances in polymer-mediated signal amplification was provided. The fundamental amplification principles and underlying mechanisms was firstly discussed, followed by an overview of representative strategies and key material systems, highlighting their applications in medical diagnostics, food safety, and environmental monitoring. Finally, current challenges and future perspectives are outlined, highlighting the potential of polymer materials to drive bioelectronic detection toward higher sensitivity, integration, and intelligence.

## 2. Underlying Mechanisms of Polymer-Mediated Amplification Strategies

### 2.1. Molecular Structure–Property Relationships in Signal Amplification

The signal amplification capability of polymeric materials is intrinsically governed by their chain architecture and electronic structure at the molecular level. Understanding the quantitative relationships between molecular parameters, such as conjugation length, side-chain engineering, and doping efficiency, and macroscopic device performance is essential for rational sensor design.

The efficiency of charge transport, which fundamentally governs the sensitivity of bioelectronic sensors, depends on the degree of π-electron delocalization along the polymer backbone. Recent computational studies by Prodhan et al. [19] revealed a direct correlation between monomer sequence and intra-chain hole mobility, demonstrating that increasing the persistence length of conjugated backbones can markedly reduce energetic disorder. Complementary work by Jiang et al. [20] further quantified the localization length of charge carriers in amorphous polymers to be approximately 8–12 Å. These findings indicate that short-range structural order is sufficient to support efficient charge transport when adequate interchain connectivity is preserved.

Side-chain design is another crucial determinant of polymer performance because it simultaneously influences electronic transport and ion dynamics. Although side chains are essential for solubility, their length and polarity significantly modulate the π–π stacking distance (d_π–π_). Hopkins et al. [21] showed that extending alkyl side chains beyond six carbon atoms introduces steric hindrance that increases d_π–π_ and reduces charge mobility. In contrast, hydrophilic side chains such as ethylene glycol are indispensable for volumetric ion uptake in organic electrochemical transistors (OECTs). He et al. [22] demonstrated a quantitative balance in this design space: higher densities of polar side chains improve ionic conductivity, yet excessive swelling can disrupt the electronic percolation network and degrade device performance.

Signal amplification strength is also strongly influenced by the charge-carrier density generated through doping. Murrey et al. [23] quantified polaron concentrations in sequentially doped conjugated polymers and demonstrated that enhancing dopant–polymer miscibility approaches the theoretical limit of doping efficiency. In addition, the orientation of polymer crystallites plays a decisive role in determining the ion-injection pathway. Kim et al. [24] reported that adjusting molecular orientation relative to the direction of ion drift can alter the transient response of OECTs by several orders of magnitude, underscoring the importance of structural alignment for real-time sensing applications.

### 2.2. Charge-Transport Optimization in Polymeric Amplification Systems

Charge-transport enhancement represents a primary mechanism by which polymers amplify bioanalytical signals. Conductive polymers improve carrier mobility by tailoring electrode morphology and forming continuous transport pathways. Their delocalized π-conjugated backbones support efficient electron transfer, making them indispensable in electronic, electrochemical, and hybrid biosensing platforms [25,26].

Polymer-based amplification arises from reductions in interfacial impedance and extensions of electron-transfer pathways. Hierarchically porous structures markedly enhance electrochemical activity. The polypeptide-doped PEDOT architecture proposed by Wang et al. [27] significantly increases the effective electroactive surface area, enabling more efficient electron exchange. Additional signal enhancement is achieved by integrating conductive polymers with metallic nanoparticles. PEDOT: PSS/AuNP composites exhibit improved conductivity through synergistic electronic coupling, which has been applied to the ultrasensitive detection of p53 proteins [28].

**Figure 1:**
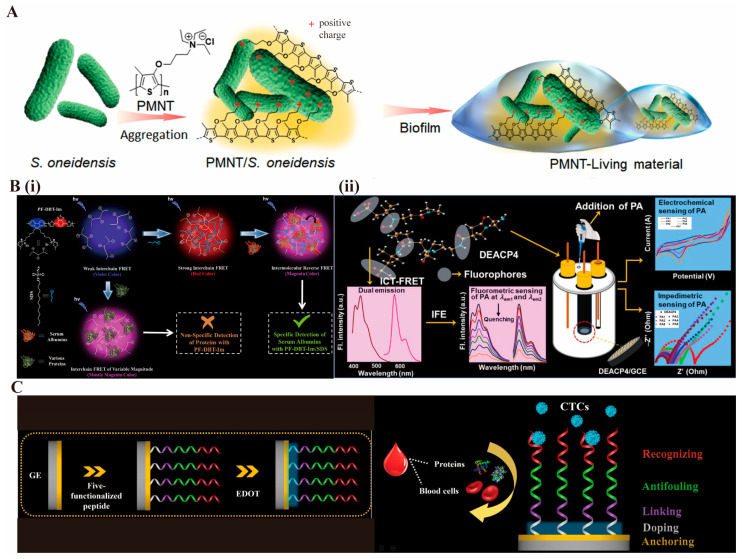
Schematic illustration of the multiple mechanisms of polymer-mediated signal amplification. The figure summarizes three core pathways through which polymeric materials enhance signal output in biosensing applications. (**A**) Charge transport optimization: Schematic carton diagram for constructing PMNT-Living material of PMNT/S. oneidensis biofilm [29]. Copyright 2022 The American Association for the Advancement of Science. (**B**) Energy transfer enhancement: (i) FRET-based amplification achieved through a design strategy for SA-specific detection using self-assembled PF-DBT-IM/SDS complexes [30]. Copyright 2022 American Chemistry Society. (ii) Dual FRET-ICT mechanism, electro/ICT-FRET active dual-state emissive aliphatic conducting polymers (DEACP) for highly sensitive detection of picric acid [32]. Copyright 2025 American Chemistry Society. (**C**) Interfacial stability improvement: Antifouling electrochemical biosensor for direct detection of circulating tumor cells (CTCs) in blood [33]. Copyright 2022 American Chemistry Society.

Polymeric materials also facilitate electron transfer across biotic–abiotic interfaces. Cationic polymers were shown by Wang et al. [29] to electrostatically assemble with biofilms, thereby promoting transmembrane electron transport and amplifying microbial metabolic signals. The high conductivity of PMNT supported efficient electron transfer within the biofilm, resulting in marked amplification of electrochemical output (Figure 1A). These studies collectively emphasize that conductive and conjugated polymers can establish continuous conduction networks and high-surface-area hybrid interfaces that dramatically strengthen electrochemical sensing performance.

### 2.3. Energy-Transfer Enhancement Through Polymer-Assisted Optical Processes

In addition to electronic transport modulation, polymeric materials enhance optical signal output through tailored energy-transfer processes. Conjugated polymers can act as efficient energy donors or acceptors within Förster resonance energy transfer (FRET) systems, enabling substantial fluorescence modulation.

Hussain et al. [30] developed a conjugated-polymer/surfactant self-assembled FRET probe capable of detecting serum albumin with fluorescence recovery up to 40-fold (Figure 1B(i)). Although UV excitation introduced background interference, later studies overcame this limitation. Tang et al. [31] incorporated upconversion nanoparticles with thermoresponsive PNIPAm carriers to create a near-infrared ratiometric sensing system that effectively eliminated autofluorescence and achieved recovery efficiencies exceeding 97%. Extending this concept, Sanfui et al. [32] designed dual FRET–ICT polymer probes for picric acid detection, reaching nanomolar detection limits in both aqueous and organic media (Figure 1B(ii)). These optical platforms demonstrate how polymer structures enable precise tuning of donor–acceptor distances, mitigation of background noise, and substantial enhancement of excitation–emission efficiency.

### 2.4. Interfacial Stability and Antifouling Regulation by Polymeric Materials

Interfacial stability is a fundamental requirement for achieving reliable signal amplification in biological fluids and complex environmental matrices. Polymeric materials improve interface robustness through antifouling behavior, hydration regulation, and mechanical reinforcement.

Han et al. [33] introduced a multifunctional peptide-PEDOT biosensor capable of selective circulating tumor cell recognition while maintaining high signal fidelity (Figure 1C). The electrodeposited PEDOT layer enhanced electron transfer and minimized nonspecific adsorption, significantly improving the signal-to-noise ratio. Geng et al. [13] developed a dual-conductive MXene-PEDOT: PSS hydrogel biosensor for the ultrasensitive detection of carcinoembryonic antigen. The hydrophilic MXene framework exhibited excellent antifouling capability, while PEDOT: PSS reinforced both conductivity and structural stability.

These examples highlight how polymeric architectures act as adaptive interfacial regulators capable of reducing signal drift, maintaining electron-transport continuity, and ensuring accurate biomolecular recognition under diverse sensing conditions.

Collectively, the studies summarized above demonstrate that polymeric materials enhance sensing performance through a combination of molecular-level structural control, optimized charge-transport pathways, regulated energy-transfer processes, and strengthened biointerfaces. These multidimensional amplification mechanisms allow polymers to simultaneously improve sensitivity, specificity, and operational stability. As molecular design strategies continue to advance, polymer-mediated amplification is expected to play an increasingly central role in enabling highly sensitive and reliable biosensing even in complex and dynamic environments.

## 3. Typical Polymer-Mediated Signal Amplification Strategies

In bioelectronic detection, the key to signal amplification lies in constructing efficient electron and ion transport pathways and accurately converting molecular recognition events into measurable signals. Due to their programmable molecular structures and designable interfacial properties, polymeric materials have emerged as ideal platforms for multidimensional signal amplification. Among them, CPs serve as both the electronic backbone and the signal bridge, playing a fundamental “underlying conductor” role in various amplification strategies. Their π-conjugated backbone and reversible redox characteristics enable the formation of continuous charge-transport networks at the electrode-solution interface, thereby significantly reducing interfacial impedance and enhancing electron transfer efficiency [34,35].

Beyond their intrinsic electrical advantages, the high interfacial compatibility of CPs further enables flexible integration with diverse functional systems, laying the foundation for constructing multilevel amplification architectures. This versatility allows for the customization of amplification performance to meet specific biosensing requirements, as illustrated by several representative hybrid systems below. For instance, combining conducting polymers with MIPs introduces molecular recognition and enrichment capabilities while maintaining high charge mobility of CPs [36,37,38]. Meanwhile, the formation of conducting polymer nanocomposites (CPNs) can further enhance conductivity and catalytic activity [39,40]. When integrated into hydrogels or three-dimensional porous networks, these CPs-based systems achieve an optical balance between flexibility, permeability, and large surface area, making them particularly suitable for wearable and implantable biosensing applications [41,42,43]. Furthermore, recent studies have explored the integration of CPs with bioinspired systems, overcoming the limitations of conventional devices in dynamic physiological environments [44]. Such bioelectrically active interfaces not only structurally resemble natural membrane systems but also enable high-fidelity electrochemical signal transduction and amplification, offering new design insights for flexible and biocompatible bioelectronic devices [45]. In addition, coupling CPs with stimuli-responsive polymers yields systems that can dynamically respond to external cues (e.g., pH, temperature, light, or electric fields) and achieve self-regulated signal amplification. By introducing responsive groups or dopant ions into the polymer backbone, these materials realize adaptive signal amplification and feedback control, endowing sensing systems with intelligent and self-tuning capabilities [46,47,48].

Overall, CPs serve as a unifying foundation across various signal amplification strategies, functioning not only as the backbone for electronic transport but also as a bridge for interfacial energy and information coupling. To provide a comprehensive understanding of polymer-mediated signal amplification, this review categorizes polymeric materials based on their dominant functional roles and structural architectures in the sensing interface. Although hydrogels and molecularly imprinted materials are intrinsically polymers, they are discussed as distinct categories here due to their unique amplification mechanisms: MIPs serve primarily as synthetic recognition elements for specific target capture; Functional Polymer–Nanocomposite Platforms leverage synergistic effects between polymer matrices and nanomaterials for multimodal signal enhancement; Polymeric Hydrogels are highlighted for their three-dimensional porous scaffolds that facilitate biomolecule immobilization and mass transport; Functional Biointerfaces mimic biological systems to optimize interfacial compatibility; and Stimuli-Responsive Polymers enable dynamic, adaptive signal modulation. This classification framework allows for a focused discussion on how different polymer engineering strategies contribute to the core goals of sensitivity enhancement and noise reduction (Table 1).

### 3.1. Signal Amplification Mediated by MIPs

MIPs are synthetic recognition materials that incorporate template molecules during polymerization to create specific binding cavities complementary in size, shape, and functionality to the target analyte. After template removal, these polymers can selectively capture and amplify trace targets within complex matrices, enabling highly sensitive and specific detection [49]. Compared with natural recognition elements such as antibodies or enzymes, MIPs exhibit superior stability, resistance to harsh environmental conditions, and excellent reusability [50]. Their amplification capability arises from two synergistic effects: the enrichment of analytes through high-affinity and high-density recognition sites, which significantly enhances local target concentration at the sensing interface, and the integration of MIPs with advanced signal transduction materials to construct unified “recognition-amplification” sensing architectures that convert molecular binding events into amplified electrical or optical outputs [51].

In the field of electrochemical sensing, the integration of MIPs with nanoconductive materials has proven highly effective in enhancing electron transfer efficiency and constructing synergistic signal amplification interfaces. Li et al. [52] developed a stable electrochemical sensing platform for detecting phenoxyacetaldehyde (PEF) by electropolymerizing a polypyrrole-based MIP film onto a black phosphorus nanosheet (BPNS) and gold nanoparticle (AuNP)-modified electrode. The BPNS/AuNP composite substrate not only greatly increased the electrode’s surface area but also facilitated electron transport, providing an excellent electrochemical signal background, while the MIP coating ensured high selectivity and structural stability. The sensor demonstrated high sensitivity in complex food matrices such as milk and orange juice, highlighting the effectiveness of rationally designed heterostructures in overcoming the intrinsic limitations of MIP-based sensors. Similarly, Alam et al. [53] introduced amyloid fibrils (AFs) into an MIP framework to fabricate an electrochemical impedance sensor for ultrasensitive tryptophan (Trp) detection. The large surface area and abundant functional groups of AFs enhanced molecular recognition efficiency and amplified interfacial resistance variations upon target binding, enabling the sensor to achieve exceptional sensitivity (LOD lower to 8 pM) under non-Faradaic EIS measurement conditions. The results obtained from milk and cancer cell culture medium were highly consistent with those from HPLC analysis, demonstrating the feasibility and potential of MIP-AF composite sensors for selective detection of small-molecule metabolites in complex biological environments.

Beyond electrochemical sensing, the integration of MIPs with electrochemiluminescence (ECL) technology has gained significant attention in recent years as an emerging and powerful signal amplification strategy, leveraging the inherent high sensitivity of ECL with the specific recognition of MIPs. For instance, Cao et al. [54] developed an ECL sensor for creatinine detection by combining a PANI hydrogel, MIP, and a self-enhanced ECL system, achieving highly sensitive and selective detection of creatinine in human serum and urine samples. Zhang et al. [55] employed perovskite quantum dots coated with a molecularly imprinted silica layer (MIP/CsPbBr_3_-QDs) as both recognition and response elements to construct a highly efficient MIP-based ECL sensor for the detection of prometryn in environmental and biological samples. Similarly, Li et al. [56] fabricated a molecularly imprinted electrochemiluminescent sensor (MIECLS) for ultra-trace detection of human serum albumin (HSA) using a click-reaction-mediated labeling approach, achieving exceptional sensitivity and selectivity. These “recognition-modulated” rather than “directly labeled” strategies demonstrate a new paradigm for background-free, ultra-sensitive MIP-based sensing. Collectively, these advances highlight the growing role of MIPs as versatile and robust alternatives to antibodies, offering high specificity and signal enhancement for complex analytical environments.

In summary, MIPs offer superior specificity and stability compared to biological receptors, making them ideal for trace detection in harsh environments. However, challenges such as slow binding kinetics, potential template leakage, and heterogeneous binding site distribution still limit their broader commercial application.

### 3.2. Functional Polymer–Nanocomposite Platforms for Multimodal Signal Amplification

The integration of functional polymers with nanomaterials represents one of the most rapidly evolving directions in polymer-mediated signal amplification strategies. These hybrid systems exploit the synergistic combination of the programmable functionality of polymers with the high conductivity and large surface area of nanomaterials, enabling interfacial-level cooperative signal amplification. The amplification mechanisms can be summarized as follows: (i) construction of efficient electron/ion transport channels to minimize interfacial impedance; (ii) introduction of bifunctional sites that provide both molecular recognition and catalytic activity; and (iii) modulation of heterostructures to achieve multidimensional amplification of optical, electrochemical, and chemical signals. Such hybrid systems not only enhance sensitivity and selectivity but also improve stability and multimodal responsiveness under complex detection conditions.

To elaborate on the practical implementation of these mechanisms, the integration of functional polymers with nanomaterials has emerged as an effective route to achieve multi-level signal amplification in bioelectronic sensing. By coupling molecular recognition with enhanced charge or energy transfer, these hybrid systems can simultaneously boost sensitivity and stability. For instance, Wang et al. [57] demonstrated a molecularly imprinted electrochemiluminescent (MIP-ECL) sensor incorporating Au@Cu:ZIF-8 nanocomposites, achieving ultrasensitive detection of malathion (LOD = 0.18 pg/mL) with a broad linear range and excellent recovery in agricultural samples. This work exemplifies the synergistic coupling between metal–organic frameworks (MOFs) and noble metals, which effectively amplifies luminescent signals through accelerated electron transfer and enhanced recognition efficiency.

**Figure 2:**
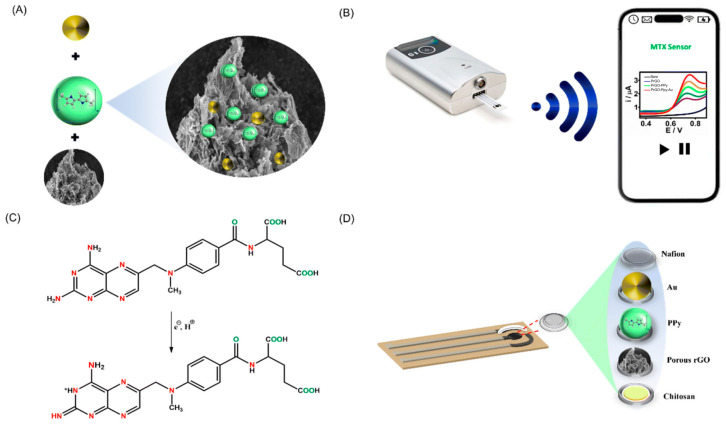
Diagrams showing the synthesis and sensing process. (**A**) The PrGO-PPy-Au nanocomposite. (**B**) Recording voltammograms of methotrexate using a Bluetooth-enabled handheld potentiostat and a mobile device. (**C**) Electrochemical oxidation behavior of methotrexate. (**D**) Diagram depicting the modified SPCE sensor’s layered architecture incorporating a PrGO-PPy-Au composite within a chitosan matrix and protected by a Nafion layer [59]. Copyright 2022 The American Association for the Advancement of Science.

Building upon this concept of interfacial synergy, researchers have further explored diverse nanomaterial scaffolds to tailor amplification performance for specific sensing scenarios. Wu et al. [58] fabricated a PANI-AuNP composite electrode, where the conductive PANI backbone and catalytically active AuNPs formed a continuous charge transport network, amplifying the oxidation current of Cd^2+^ by more than 300%. Such CP-metal nanocomposite systems highlight the crucial role of interfacial electron mobility in voltammetric signal enhancement. Extending this strategy to carbon-based scaffolds, Sukumaran et al. [59] developed a PrGO-Au-PPy hybrid electrode that markedly improved charge transfer in screen-printed carbon electrodes (SPCEs), enabling continuous, high-sensitivity monitoring of methotrexate (Figure 2). The porous carbon framework not only increased the active surface area but also provided efficient ionic/electronic transport pathways, further reinforcing the concept that hierarchical polymer–nanocomposite architectures can integrate recognition, conduction, and amplification within a single sensing interface. Similarly, Younis et al. [60] designed a ternary polyindole/Ag/rGO nanocomposite that combined the high-conductivity polymer matrix, catalytic Ag nanoparticles, and the large surface area of rGO, collectively amplifying the oxidation signal of ascorbic acid with superior sensitivity and selectivity.

Beyond traditional rigid sensing platforms, polymer–nanocomposite systems have also shown great promise in flexible sensing applications, addressing the demand for wearable and implantable devices. Lawaniya et al. [61] fabricated a cost-effective flexible ammonia sensor based on nitrogen-doped carbon nano-onion/polypyrrole nanocomposites, achieving room-temperature operation with excellent response stability. Furthermore, Dehghan-Manshadi et al. [62] developed a flexible capacitive creatinine sensor by electrodepositing polyhedral polyvinylpyrrolidone/CuO and Fe_2_O_3_ nanorod structures onto a 3D TiO_2_-V_2_O_5_-PPy nanocomposite substrate. Collectively, these studies demonstrate the broad applicability of polymer–nanocomposite systems in next-generation sensing devices, where multi-component heterostructures enable high conductivity, tunable functionality, and exceptional mechanical flexibility, paving the way for high-performance chemical and biosensing applications.

In summary, functional polymer–nanocomposite systems integrate the structural programmability of polymers with the exceptional electrical, optical, and catalytic properties of nanomaterials, enabling a systematic leap from simple interfacial optimization to multimodal signal amplification. The integration of metallic, metal–organic framework, and carbon-based components not only enhances charge transport and energy transfer efficiency but also boosts the overall stability and selectivity of the sensing interface. Meanwhile, the intrinsic flexibility and processability of polymers empower these hybrid architectures to perform effectively in wearable and complex environmental sensing scenarios. Overall, polymer–nanocomposite platforms successfully integrate the conductivity of polymers with the catalytic and optical properties of nanomaterials, achieving significant signal enhancement. Nevertheless, the complexity of synthesizing uniform heterostructures and the potential aggregation of nanomaterials within the polymer matrix remain critical issues affecting sensor reproducibility.

### 3.3. Polymeric Hydrogels and Three-Dimensional Porous Networks

Polymeric hydrogels and three-dimensional (3D) porous network materials have emerged as important signal amplification platforms in bioelectronic detection owing to their highly tunable porosity and superior molecular enrichment capability. Conductive polymer hydrogels (CPHs), a unique class of soft conductive materials, integrate the advantages of hydrogels and conjugated polymer conductors [63], showing great potential for bioelectronic applications. Unlike 2D planar interfaces, these 3D architectures offer a dual amplification mechanism: their highly porous structure significantly increases the specific surface area for high-density biomolecule loading, while their tunable mesh size facilitates the efficient diffusion of ions and substrates [64].

**Figure 3:**
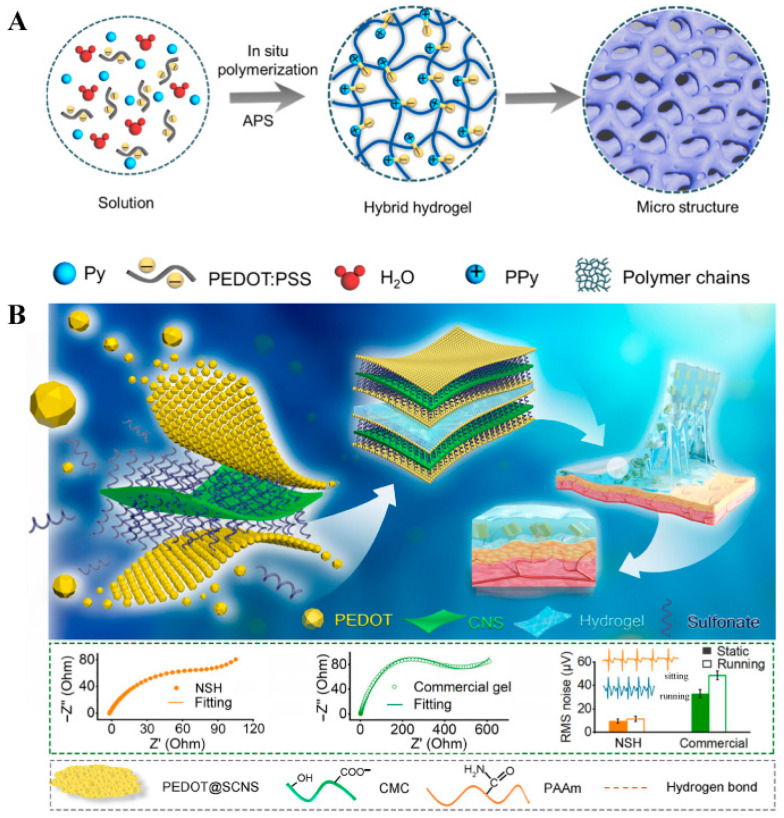
(**A**) Schematic illustration of the fabrication and structure of the PPy-PEDOT:PSS hybrid hydrogel, forming a highly conductive 3D network that effectively amplifies electrochemical current signals [65]. Copyright 2021 American Chemistry Society. (**B**) Conductive nanosheet-based microcapacitor array: the series-parallel microcapacitor network amplifies mechanical strain signals, enabling low-impedance and high-fidelity electrophysiological detection [69]. Copyright 2025 Wiley.

Constructing efficient charge and mass transport pathways within 3D networks is the fundamental strategy for amplifying bioelectronic signals. High conductivity is often achieved by assembling conjugated polymers into hydrogel frameworks. Ren et al. [65] achieved the electrostatic assembly of polypyrrole (PPy) and PEDOT:PSS to construct a hybrid hydrogel with ultrahigh conductivity (867 S m^−1^) (Figure 3A). The resulting efficient electron-transport network markedly amplified the current response in electrochemical detection. Building upon this conductivity optimization, Tang et al. [66] demonstrated that a rationally designed 3D porous framework could synergistically enhance bioelectrocatalytic signals by not only enriching the local concentration of enzymes (e.g., glucose oxidase) but also facilitating the rapid diffusion of substrates. This comparison highlights a key design principle: optimal signal amplification requires a balance between the density of conductive pathways and the permeability of the porous network. Beyond enhancing sensitivity, improving signal reliability through multi-analyte correlation and interference mitigation represents a more advanced level of amplification logic. In complex physiological fluids like sweat, signal drift caused by environmental fluctuations (e.g., pH changes) often compromises detection accuracy. To overcome this, Xu et al. [67] developed a TA-Ag-CNT-PANI composite hydrogel capable of simultaneous tyrosine and pH monitoring. By utilizing the real-time pH signal to calibrate the tyrosine response in situ, this system transforms “passive signal output” into “active accuracy enhancement,” effectively amplifying the signal-to-noise ratio in dynamic environments. This approach signifies a transition in hydrogel-based sensing from single-parameter detection to intelligent, self-calibrated analysis. Li et al. [68] addressed signal integrity through a sweat-resistant bioelectronic sensor. Its strong wet adhesion maintained robust skin-sensor contact under dynamic conditions, preventing signal interruption. Additionally, introducing MXene nanosheets induced an entropy-to-enthalpy transition of ion transport under strain, significantly enhancing strain sensitivity and enabling mechanical-to-electrical signal amplification for physiological motion monitoring. From a more integrative perspective, Shi et al. [69] pushed conductive hydrogels’ signal amplification capacity to a new dimension by designing a dual-mode platform that functions as both a “mechanical signal amplifier” (capacitive mode) and a “high-fidelity electrophysiological signal collector” (electrode mode) (Figure 3B). The series-parallel network of conductive nanosheet-based microcapacitors amplified electromechanical responses to strain, while the hydrogel’s superior skin conformity and low impedance further boosted the signal-to-noise ratio of recorded EMG, ECG, and EEG signals.

These diverse examples collectively illustrate that conductive hydrogels achieve efficient signal amplification through multi-dimensional and multi-mechanistic cooperation. By integrating electron/ion transport optimization, structural compliance, and interfacial coupling, polymeric hydrogel systems provide a robust platform for high-fidelity signal acquisition and amplification in bioelectronics.

Despite these compelling advancements, conductive hydrogels still face several critical challenges hindering their large-scale commercialization in bioelectronics. First, inherent trade-offs exist among key performance parameters, such as mechanical strength/toughness, electrical conductivity, and sensitivity, which are difficult to optimize simultaneously [70]. Second, under complex physiological conditions (e.g., sweat, dynamic mechanical loads), long-term electrochemical stability and signal drift issues remain unresolved. Finally, as integral components of electronic systems, establishing stable interfacial integration between hydrogels and rigid circuits or power modules continues to be a major bottleneck for reliable wearable devices. Future research should focus on elucidating molecular-level “structure–property–function” relationships to overcome these limitations and accelerate the translation of conductive hydrogels from laboratory research to real-world applications.

In conclusion, while conductive hydrogels provide excellent biocompatibility and high surface area for biomolecule loading, their application is often constrained by a trade-off between mechanical toughness and electrical conductivity. Furthermore, the swelling behavior of hydrogels in physiological fluids can lead to structural instability and signal drift over long-term use.

### 3.4. Functional Biointerfaces Based on Conducting Polymers

Incorporating CPs into biomimetic systems offers significant opportunities for advancing bioelectronic interfaces. However, challenges such as inadequate membrane electrical sealing and long-term stability remain major obstacles, limiting their applicability in mimicking natural or synthetic membrane environments. To address this bottleneck, Schafer et al. [45] constructed a droplet-polymer bilayer (DPB) composed of lipid-coated aqueous droplets interfaced with the high-performance conducting polymer poly(3,4-ethylenedioxythiophene):polystyrene sulfonate (PEDOT:PSS). This approach enables the controlled assembly of diverse lipid compositions and provides superior electrical sealing compared with conventional supported lipid bilayers (SLBs), thereby establishing a robust platform for integrating bioelectronics with synthetic, natural, or hybrid lipid membrane systems.

Beyond lipid membrane optimization, the combination of conducting polymer hydrogels with biomimetic architectures has emerged as another effective strategy to overcome traditional interfacial engineering limitations, revealing great potential for biohybrid sensing applications. Li et al. [71] systematically elucidated how conducting polymer hydrogels enhance multimodal signal transduction in artificial skin sensors by optimizing coupled ionic and electronic transport pathways. Building on this, Yuan et al. [72] further demonstrated that integrating the conducting polymer PVDF with a fish lateral-line-inspired curvature structure can effectively concentrate mechanical stimuli and amplify weak respiratory signals, highlighting the synergistic advantage of structural biomimicry and polymer-mediated signal amplification.

In the construction of bioinspired interfaces, conductive hydrogels exhibit unique and irreplaceable advantages. Their three-dimensional hydrophilic networks serve as flexible supports for artificial membranes, while the embedded conductive components enable efficient signal transduction and amplification. For instance, certain ionically conductive hydrogels exhibit mechanical moduli comparable to those of native tissues, and their ion transport mechanisms closely resemble biological electrochemical signaling, making them ideal platforms for mimicking neural-skin interfaces [16]. Furthermore, Chen et al. [73] developed a biomimetic sensor based on a carbon nanomaterial/conducting polymer composite, which emulated the arched geometry of fingerprints to achieve an ultrawide strain range (0–190%), high linearity (R^2^ = 0.996), and integrated temperature sensing capability. This work demonstrates that the synergy between macrostructural biomimicry and nanoscale composite design can simultaneously enhance both skin-like multimodal perception and signal amplification.

Therefore, integrating conducting polymer hydrogels with biomimetic membranes, skin-like receptor architectures, or neural interfaces offers a promising pathway to overcome impedance mismatch and signal attenuation issues in conventional bioelectronic systems. Such integration could further enable the development of intelligent biomimetic platforms with intrinsic biocompatibility, multimodal sensing, and closed-loop feedback capabilities. This convergence is progressively blurring the boundary between “biological” and “electronic,” laying the foundation for seamless bio-machine integrated sensing and interaction. Conductive polymer-based biointerfaces effectively bridge the gap between hard electronics and soft biological systems, minimizing impedance mismatch. However, constructing stable, long-lasting interfaces that resist delamination and maintain electrical performance in dynamic wet environments remains a significant engineering challenge.

### 3.5. Adaptive and Stimuli-Responsive Polymers

Stimuli-responsive polymers represent a cutting-edge direction in polymer-mediated signal amplification. These materials can undergo reversible conformational or functional changes upon external stimuli, such as temperature, pH, humidity, light, electric fields, or chemical molecules, thereby enabling dynamic regulation and “intelligent amplification” of sensing signals.

In the field of flexible and wearable sensing, stimuli-responsive polymers demonstrate exceptional adaptability and multimodal detection capabilities. Xia et al. [74] developed a monolayer Au@pNIPAm microgel sensor that exploits the thermoresponsive and strain-sensitive properties of pNIPAm for multidimensional detection of physiological pressures (sound, pulse, respiration) and metabolic markers (bacteria, uric acid), highlighting its potential for comprehensive physiological monitoring. Yi et al. [75] designed a water-responsive ultra-contractile polymer (WRAP) electrode array that rapidly deforms upon hydration, achieving intimate tissue adhesion for high signal-to-noise, low-damage neural recording. Ma et al. [76] reported a multifunctional POM-ionic liquid conductive hydrogel capable of dual responses to mechanical and chemical stimuli (HCl/NH_3_, Fe^3+^/EDTA^2−^), integrating strain sensing, reversible fluorescence switching, and antimicrobial functions, thereby offering a novel platform for wearable biosensing and information encryption.

**Figure 4:**
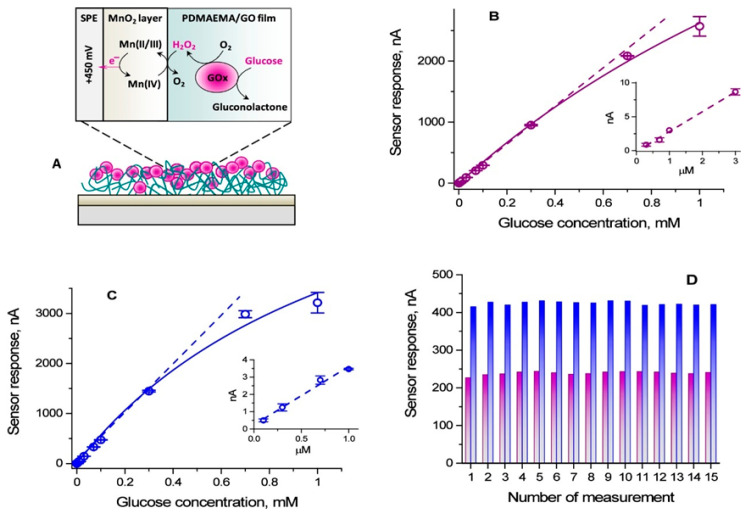
(**A**) Principle of amperometric detection of β-d-glucose; (**B**,**C**) dependences of the enzymatic (amperometric) responses on the β-d-glucose concentration for the SPE/MnO_2_/PDMAEMA (25 °C)/GOx (**B**) and SPE/MnO_2_/PDMAEMA (40 °C)/GOx (**C**) constructs; (**D**) operational stability of the enzymatic (amperometric) responses measured for 0.1 mM of β-d-glucose. Solid lines through the experimentally derived data points are drawn only as guides to the eye. The dashed lines represent linear regression fits [77]. Copyright 2025 American Chemistry Society.

**Figure 5:**
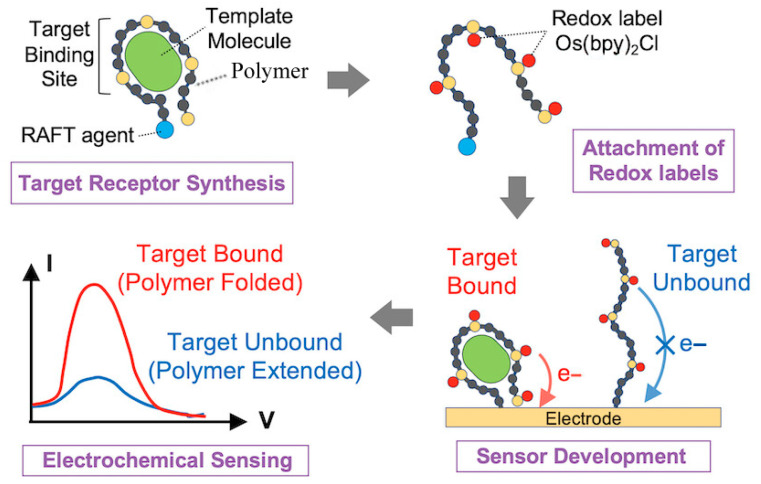
A target-selective biosensing platform based on a stimuli-responsive polymer. In this system, a linear polymer with specific affinity toward the target molecule is synthesized via RAFT polymerization using the target as a template, followed by the introduction of redox-active groups (Os(bpy)_2_Cl) after template removal. Upon target binding, the polymer undergoes a conformational transition from an extended to a folded state, bringing the redox centers closer to the electrode surface and thereby accelerating electron transfer. Voltammetric measurements reveal a target concentration-dependent signal response, demonstrating the unique capability of stimuli-responsive polymers for dynamic molecular recognition and electrochemical signal amplification [79]. Copyright 2024 American Chemistry Society.

Beyond flexible wearable devices, stimuli-responsive polymers also exhibit unique advantages in electrochemical sensing, where their tunable responsiveness enables precise regulation of detection performance. Sigolaeva et al. [77] developed a PDMAEMA-based dual-responsive polymer-enzyme composite membrane, where enzymatic activity and electron transfer rates are modulated by pH and temperature, achieving tunable sensitivity (47–68 μA/(mM·cm^2^)) and submicromolar glucose detection (Figure 4). Mutharani et al. [78] fabricated a PVCL/PPY semi-interpenetrating conductive microgel for reversible temperature-switchable detection of the nitrogen mustard drug chlorambucil. Ahmadian-Alam et al. [79] designed a redox-labeled polymer system, poly(NIPAM-VP-MAA)-g-Os(bpy)_2_Cl (Figure 5), capable of ultrasensitive detection of non-electroactive neurotransmitters such as glutamate and histamine, exhibiting dual “on/off” signal modes, excellent selectivity, and reusability. Similarly, Ding et al. [80] developed a multifunctional PNIPAAm-co-PBA/TP copolymer interface capable of detecting multiple neurochemicals (glucose, sialic acid, ATP) with high sensitivity, anti-fouling properties, and excellent controllability for in vivo applications.

Collectively, stimuli-responsive polymers achieve dynamic signal amplification through reversible structural modulation. Their programmable interfacial responsiveness provides a powerful foundation for the integration of flexible sensing, real-time biological monitoring, and next-generation intelligent bioelectronic devices. Specially, these materials offer three key advantages in signal amplification: (i) conformational modulation and phase transition enable real-time signal enhancement; (ii) adaptive material interfaces improve the coupling efficiency of biological recognition events; and (iii) intrinsic responsiveness endows the system with reversibility and self-regulation. Looking forward, integrating stimuli-responsive polymers with CPs, electroactive hydrogels, or biomimetic membranes may enable multi-stimuli coupled responses and cross-interface signal amplification. This integration is expected to drive the evolution of intelligent bioelectronic platforms from “responsive” to “feedback-regulated” systems, opening new avenues for advanced bioelectronic detection and monitoring. Despite this advantage, these systems often suffer from slow response times and hysteresis during reversible cycles. Additionally, ensuring specificity in complex environments where multiple interfering stimuli may coexist is a key hurdle for future development.

## 4. Applications of Polymer-Mediated Signal Amplification in Bioelectronic Sensing

With the gradual maturation of these mechanisms, polymer-mediated signal amplification has transitioned from theoretical exploration to practical implementation, demonstrating remarkable application potential across multiple domains. In practical applications, polymeric materials, characterized by their high sensitivity, excellent selectivity, and strong environmental adaptability, have become vital mediators of signal amplification in bioelectronics and have further evolved into essential building blocks for interdisciplinary sensing platforms. Based on these characteristics, this section will focus on recent advances and future trends of polymer-assisted signal amplification in biomedical diagnostics and health monitoring, food safety assessment, and environmental surveillance. Particular attention will be given to differences and optimization strategies in target recognition, signal transduction, and interfacial stability, aiming to identify viable pathways for translating polymer-based amplification strategies toward clinical and field-ready applications.

### 4.1. Medical Diagnosis and Health Monitoring

In medical diagnostics, polymer-mediated signal amplification strategies have attracted considerable attention due to their high sensitivity, tunable interfaces, and excellent system compatibility. In the field of nucleic acid detection, the primary challenge lies in balancing ultra-high sensitivity with stability in complex biological fluids. Conductive polymers have been engineered to address this trade-off through diverse structural strategies. For instance, Zhao et al. [81] developed an rGO transistor array integrated with a PAA/PEDOT composite layer. This design not only improves hole mobility but also provides carboxyl anchoring sites for covalent immobilization of Y-shaped DNA probes. The resulting three-dimensional DNA architecture induces more pronounced charge perturbations during hybridization, achieving a femtomolar-level detection limit. However, for clinical applications involving plasma, interfacial fouling becomes a limiting factor. Addressing this, Song et al. [82] engineered a BSA/polyA_8_ antifouling interface on a solution-gated graphene transistor (SGGT). The dense hydration layer formed by this interface effectively blocked nonspecific adsorption, enabling ultra-sensitive detection of exosomal miRNA-196a in clinical plasma (~10^−19^ M, AUC = 0.98). The performance outperforms conventional serum biomarkers such as CA19-9. Further pushing the boundaries toward point-of-care testing (POCT), Zhang et al. [83] integrated two-dimensional metal carbides (MC) with carbon nanotubes (CNT) to construct a miniaturized FET biosensor. The MC@CNT heterojunction offered a large surface area and rapid electron transfer channels, enhancing transconductance by 42% and enabling non-invasive detection of exosomal miRNA-122 in urine. This advancement paves the way for the development of point-of-care (POCT) testing platforms.

While nucleic acid sensors prioritize amplification of hybridization events, protein detection demands robust recognition interfaces capable of operating in dynamic physiological environments. Thus, signal amplification in protein detection places greater emphasis on microenvironmental control and anti-interference design [84]. Ting et al. [85] developed a PANI-based MIP electrochemical sensor for ultra-trace detection of bovine serum albumin (BSA), achieving a limit of detection (LOD) of 2.3 pg/mL. Notably, acetic acid–sodium dodecyl sulfate (AcOH-SDS) elution was introduced to simultaneously improve electron transport, endowing the sensor with both high sensitivity and low cost. Zheng et al. [86] designed an organic electrochemical transistor (OECT) microelectrode array, which can simultaneously detect four Alzheimer’s disease-related proteins with zeptomolar-level sensitivity and 100% clinical classification accuracy. To enhance biointerface stability, Zhang et al. [87] incorporated zwitterionic PEDOT-PC into both the gate and channel regions of the sensor, enabling real-time detection (60 s, 0.11 pg/mL) without the need for a blocking step. Similarly, Li et al. [88] introduced stapled-peptide-based antifouling interfaces to resist enzymatic degradation, yielding CEA detection results consistent with standard assays. Collectively, these studies highlight the synergistic advancement of CPs in electron transport optimization, molecular imprinting recognition, and antifouling engineering, driving protein detection from single-parameter sensitivity enhancement toward integrated multifunctional performance.

Beyond static diagnostic testing, polymer systems have also shown new promise in continuous health monitoring, particularly for tracking metabolic indicators such as pH, glucose, and lactate. Qian et al. [89] optimized the electronic structure and proton-doping capacity of polyaniline via donor–acceptor molecular engineering, achieving a sweat pH sensitivity of 65.193 mV/pH and improving the sensor’s stability by 3.6–9.0 times. Building on this, Li et al. [90] and Zhang et al. [91] advanced the field by integrating sensing modules into fiber-based architectures. These “all-in-one” stretchable fibers not only withstand significant mechanical strain but also enable the simultaneous monitoring of multiple metabolic indicators (e.g., glucose, lactate, electrolytes), paving the way for next-generation smart textiles that seamlessly integrate into daily life.

Despite significant progress, medical applications of polymer-based amplification technologies still face substantial challenges, particularly concerning long-term interface stability in vivo. Complex biological environments such as blood, sweat, and interstitial fluids contain abundant proteins and metabolites that tend to adsorb onto sensor surfaces, resulting in biofouling, signal drift, and compromised sensitivity during continuous operation. In addition, the mechanical mismatch between rigid electronic components and soft tissues can induce inflammation, discomfort, or device delamination, thereby limiting the reliability of long-term monitoring. Nevertheless, polymer-mediated amplification strategies are driving clinical biosensing systems beyond traditional single-point, high-sensitivity assays toward continuous, flexible, and multidimensional information acquisition. By integrating electron–ion cooperative transport, antifouling interface engineering, and structurally adaptive polymer designs, recent advances have simultaneously improved detection sensitivity and operational stability across nucleic acid, protein, and metabolic monitoring. Representative studies summarized in Table 2 illustrate how these materials are transitioning from simple “diagnostic carriers” to intelligent biointerfaces capable of maintaining signal fidelity under physiological conditions. This technological evolution expands the applicability of bioelectronics and builds a practical foundation for bridging personalized health management with next-generation wearable and implantable diagnostics.

### 4.2. Food Safety Monitoring

Notably, polymer-based sensing technologies have not only achieved remarkable progress in medical diagnostics but also are rapidly expanding into critical public domains such as food safety monitoring. This cross-disciplinary expansion not only underscores the universality of these technologies, but also reflects the growing societal demand for rapid, convenient, and reliable analytical tools. In food testing, however, achieving specific recognition and stable signal output within complex sample matrices remains a core technical challenge for their practical application.

To address this challenge, researchers have made significant breakthroughs in detecting different types of food contaminants, starting with pesticide residues and antibiotics. As biomimetic recognition materials, MIPs enable highly selective capture of trace analytes from complex environments. Lakavath et al. [92] developed an electrochemical sensing platform by integrating MIPs with reduced graphene oxide and polypyrrole, achieving efficient electron transport and strong molecular selectivity for rapid, real-time detection of atrazine. Across various agricultural products, the system exhibited excellent recovery and reproducibility. For wearable and on-site applications, Paschoalin et al. [93] fabricated an enzyme-free flexible sensor by combining a polylactic acid (PLA) fiber substrate with screen-printed electrodes, realizing in situ and nondestructive detection of pesticide residues directly on the surface of fruits and vegetables. This structural innovation marks a key step in advancing polymer sensing systems from laboratory testing to portable, field-deployable tools. Further, Yuan et al. [94] introduced Bi_2_WO_6_ quantum dot/covalent organic framework (COF) heterojunctions into MIP-based systems. By leveraging synergistic effects between photogenerated carriers and donor–acceptor interactions to achieve femtomolar-level detection of methyl parathion, they demonstrated the powerful potential of photoelectrochemical–MIP hybrid amplification. Similarly, Adane et al. [95] designed an electrochemical sensor based on a thermally annealed Au-Ag alloy nanoporous substrate, combined with functionalized carbon nanotubes and poly-L-serine. This sensor enables simultaneous detection of sulfathiazole (SFT) and sulfamethoxazole (SFM) in honey, beef, and egg samples with picomolar sensitivity. This study revealed the critical role of Au-Ag/CNT hybridization in enhancing electrocatalytic activity and charge transfer efficiency.

In addition to organic residues, heavy metal contamination poses a persistent threat to food safety. Polymer-based sensors have shown great promise in this area by combining enrichment capabilities with conformational switching strategies. For example, Hao et al. [96] utilized a DNA tetrahedron-stabilized aptamer-MIP system to ensure highly selective recognition of Pb^2+^ and Hg^2+^, effectively overcoming interference in complex seafood matrices. Similarly, Shi et al. [97] highlighted the potential of MOF-polymer composites for multi-analyte detection, achieving simultaneous monitoring of Cd^2+^, Pb^2+^, and Hg^2+^. These studies collectively illustrate that rational interface design can significantly enhance the anti-interference performance of sensors in real-world food samples.

Foodborne pathogens are another key concern for food safety, and the integration of bioelectronics with polymer-based recognition systems has greatly improved the sensitivity and intelligence of pathogen detection. For instance, Abed et al. [98] developed a pHEMA-based SERS sensor that uses hydrogen-bond-mediated capture of Gram-negative bacterial lipopolysaccharides. Through machine learning analysis, the sensor achieves highly sensitive, multiplexed pathogen identification and classification, even in complex matrices like apple juice. Moreover, Zhang et al. [99] reported a bacterium-imprinted photoelectrochemical biosensor that employs dual-mode (active/passive) detection combined with machine learning. This biosensor achieves a detection limit as low as 1.06 CFU/mL for Staphylococcus aureus and exhibits excellent anti-interference performance in beverages and dairy samples. These advances demonstrate the power of coupling polymer interface engineering with intelligent data analytics for rapid, robust food biosafety monitoring.

To summarize, Table 3 outlines representative applications of polymer-based amplification systems in food safety detection. As shown, polymers play a pivotal role in enhancing interface control, recognition selectivity, and signal amplification across diverse contaminant types. Biomimetic systems such as MIPs, when combined with CPs, MXene, or COF-based nanocomposites, have achieved ultrahigh sensitivity and excellent reproducibility in detecting pesticides, antibiotics, and heavy metal ions within complex food matrices. Meanwhile, the development of flexible, wearable, and in situ sensing platforms has driven food safety monitoring toward greater portability and real-time capability.

A unified effort in polymer-enabled sensing is steadily guiding food safety monitoring toward multi-analyte detection, high specificity, and intelligent recognition. The synergistic integration of polymers with nanomaterials, biorecognition elements, and machine-learning algorithms has markedly improved sensitivity and anti-interference performance, offering a promising route toward next-generation smart food safety systems. Despite these advances, practical deployment still faces significant challenges, particularly regarding matrix interference and cost-efficiency. Food samples are intrinsically heterogeneous, containing abundant fats, proteins, and sugars that often necessitate labor-intensive pretreatment procedures and diminish the advantages of rapid on-site detection. Moreover, for large-scale commercialization, fabrication costs of single-use polymer-based sensors must be reduced without compromising reproducibility or robustness. Future research may therefore focus on developing antifouling and matrix-tolerant polymer interfaces, as well as reusable or regenerable sensing architectures, to bridge the gap between laboratory-level performance and real-world food monitoring applications.

### 4.3. Environmental Monitoring

Polymer-based materials have also demonstrated extensive potential in environmental monitoring. Similarly to food safety detection, the inherent complexity of environmental samples, such as fluctuating pH, high ionic strength, and coexistence of organic contaminants, poses major challenges to sensor selectivity and reliability. Thanks to their tunable molecular structures and adjustable interfacial properties, polymers have emerged as ideal candidates for developing robust and adaptive sensing platforms. In recent years, polymer-based composite sensors have been increasingly applied to the detection of heavy metal ions, organic pollutants, and gaseous contaminants, with ongoing advancements toward multifunctional, intelligent, and sustainable monitoring systems.

Water quality monitoring is a core focus of environmental sensing, and polymer-based materials have driven significant breakthroughs in this area, particularly in optimizing biofilm performance and detecting target contaminants. In terms of biofilm regulation for contamination warning and water treatment, Qi et al. [100] incorporated an appropriate amount of polypyrrole (PPy) into artificial electroactive biofilms to serve as a conductive mediator, thereby enhancing the sensitivity of Shewanella oneidensis-based biosensors for early water contamination warning and revealing the trade-off between conductivity and mass transfer. Building on this concept, Cai et al. [101] employed mild electrical stimulation to modulate the structure and electron transport efficiency of anaerobic ammonium oxidation (anammox) biofilms, achieving significantly improved nitrogen removal performance.

Beyond biological regulation, the precise detection of specific chemical contaminants remains a priority. Polymer composites have enabled high-sensitivity analysis through synergistic electrocatalysis. Maheshwaran et al. [102] developed a sensor based on polyaniline-Reactive Yellow 42 dye (Pani-RYFG) composite-modified electrodes capable of simultaneous, ultrasensitive detection of Hg^2+^ and Pb^2+^ (detection limits of 2 nM and 6.2 nM, respectively). Density functional theory (DFT) analysis further elucidated the electron transfer mechanism between metal ions and the composite matrix, confirming the system’s robustness and applicability in real water samples. In complex multi-component water analysis, Kharkova et al. [103] fabricated a dual-functional bioanalytical platform that integrates microbial consortia with nanocomposite conducting polymers, enabling simultaneous monitoring of biochemical oxygen demand (BOD) and toxicity in surface waters with enhanced sensitivity and response speed. Likewise, Jose et al. [104] employed laccase-immobilized conducting polymer electrodes for the selective detection of p-nonylphenol (PNP), achieving nanomolar sensitivity and nearly 100% recovery in real water samples. This underscores the feasibility of polymeric biosensors for monitoring endocrine-disrupting phenolic compounds.

Beyond water quality monitoring, polymer-based composites have also shown remarkable applicability in gas pollutant detection, with innovations focusing on high sensitivity, low power consumption, and flexibility. Kalyalkin et al. [105] proposed a dual-cell solid electrolyte electrochemical sensor operating at 600–700 °C for simultaneous quantification of CH_4_ and CO_2_ in biogas. The device exhibited a fast response time (<1.5 min), simple configuration, high reproducibility, and strong industrial applicability. In a complementary approach, Ramezani Farani et al. [106] designed a graphene-polyimide (Kapton)-based flexible gas biosensor, optimized via electromagnetic simulation. The device detects multiple toxic gases (e.g., CO, N_2_O, O_3_, CH_4_) through resonance frequency shifts induced by electron interactions between gas molecules and graphene, achieving high sensitivity, ultra-low power consumption (1.5 mW), and excellent mechanical flexibility, offering new opportunities for non-invasive environmental and biomedical monitoring.

Table 4 summarizes these studies demonstrating the application of polymer-based sensing systems in environmental monitoring. Recent progress highlights a clear transition from conventional single-pollutant detection toward multimodal and adaptive sensing platforms. The integration of conducting polymers, nanocomposites, and bio-recognition elements has enhanced sensitivity, selectivity, and environmental tolerance, enabling portable, wearable, and continuous on-site monitoring. Despite these advances, long-term robustness in complex and harsh environmental conditions remains a major challenge. Sensors must endure fluctuating pH, temperature variations, and biofilm-induced corrosion while maintaining stable performance. Additionally, the development of low-power or self-powered operation is essential for remote and unattended deployment. Addressing these challenges through improved interfacial stability, self-calibrating signal mechanisms, and cooperative multi-pollutant recognition strategies will be key to achieving reliable and durable polymer-based environmental sensing technologies.

## 5. Conclusions and Perspective

Polymer-mediated signal amplification has fundamentally transformed bioelectronic sensing by bridging the gap between biological recognition and electronic transduction, enabling multidimensional enhancement of sensitivity and stability. By leveraging the programmable molecular structures and tunable interfacial properties of conductive polymers, molecularly imprinted polymers, and hydrogels, researchers have developed a diverse array of sensing platforms capable of detecting trace analytes with high precision. These materials not only function as efficient electron/ion transport channels but also serve as versatile scaffolds for biorecognition and antifouling engineering, thereby significantly enhancing the sensitivity, selectivity, and stability of bioelectronic devices in medical, food, and environmental applications.

Despite these breakthroughs, the translation of lab-scale innovations to real-world applications is currently impeded by critical bottlenecks in structural stability, molecular selectivity, and system integration. Key challenges include limited structural stability under physiological conditions, insufficient molecular selectivity in complex matrices, and signal interference from non-specific background noise. Consequently, achieving an optimal balance between ultra-high sensitivity and long-term reliability, while developing self-calibrating and antifouling interfacial systems with superior reusability, remains a primary goal. Furthermore, the seamless integration of flexible polymer sensors with wireless communication modules, self-powered energy units, and intelligent data-processing algorithms poses a significant engineering challenge that requires coordinated design strategies.

The future of this field lies in the convergence of “intelligent” polymer chemistry with data-driven bioelectronics, moving beyond simple sensitivity improvement toward self-adaptive, integrated, and closed-loop monitoring systems. Looking ahead, the convergence of polymer chemistry with micro/nanofabrication and information technology promises to open new avenues for innovation. Next-generation polymers featuring adaptive self-regulation, biodegradability, and multi-stimuli responsiveness hold great promise for building safe, stable, and sustainable real-time monitoring platforms. Notably, this evolution will provide crucial theoretical and technological support for the interdisciplinary convergence of precision medicine, food safety, and environmental health monitoring.

## Figures and Tables

**Table 1:** Comparative Analysis of Polymer-Mediated Signal Amplification Strategies.

Strategy	Key Mechanism	Sensitivity	Stability	Cost	Primary Limitation
MIPs	Specific binding cavities enrich target molecules	High	High (Thermal/Chemical)	Low	Slow binding kinetics; Template leakage
Nanocomposites	Synergistic effect of conductivity and catalysis	Very High	Moderate	Moderate/High	Aggregation of nanofillers; Complex synthesis
Hydrogels	3D porous structure increases loading capacity	High	Moderate	Low	Trade-off between conductivity and mechanics
Biointerfaces	Mimics natural environment to reduce impedance	Moderate	Low (In vivo)	High	Long-term biological stability; Fabrication complexity
Stimuli-Responsive	Dynamic modulation of signal via phase change	High	Moderate	Moderate	Response hysteresis; Environmental cross-sensitivity

**Table 2:** Applications of Polymer-Mediated Signal Amplification Strategies in Medical Diagnosis and Health Monitoring.

Target Analyte	System Type/Material Structure	Amplification Mechanism	Key Performance Indicators	Application Scenario/Advantages	Ref.
miRNA-196a	rGO transistor array with PAA/PEDOT composite layer and Y-shaped DNA probe	Conductive polymer forms continuous charge transport channels; 3D DNA structure enhances electrostatic perturbation response	LOD ~ 10^−19^ M; AUC = 0.98	Clinical plasma samples; highly sensitive detection without nucleic acid preamplification	[82]
miRNA-122	MC@CNT heterojunction miniaturized FET	High surface area and rapid electron transfer pathways; 42% enhancement in transconductance	Fast response; noninvasive urine detection	Home-use and POCT (point-of-care testing) applications	[83]
Alzheimer’s disease-related proteins	Microelectrode array-integrated OECT (organic electrochemical transistor)	Electrochemical multi-point signal amplification; simultaneous multi-target detection	Zeptomolar-level sensitivity; 100% classification accuracy	Multiplexed, label-free detection	[86]
C-reactive protein (CRP)	OECT modified with zwitterionic conductive polymer PEDOT-PC	Intrinsic antifouling and charge-regulated interface	LOD 0.11 pg/mL; response time 60 s	Ready-to-use POCT; anti-biofouling capability	[87]
Carcinoembryonic antigen (CEA)	Stapled-peptide-modified electrochemical sensing interface	Enhanced protease resistance; improved long-term signal stability	Results consistent with clinical standards	Robust protein detection; long-term storage stability	[88]
pH (sweat)	Molecularly engineered polyaniline	H^+^ doping/de-doping-driven electrochemical signal amplification	Sensitivity 65.193 mV/pH; stability improved 3.6–9.0×	Long-term sweat monitoring; flexible wearable sensors	[89]
H_2_O_2_/Ascorbic acid	Active nanocomponents embedded in 3D PEDOT:PSS network	Structure-function integrated conductive fiber; stable signal amplification	<10% performance decay after 14 days or 1000 bending cycles	Continuous health monitoring; wearable integration	[90]

**Table 3:** Applications of Polymer-Based Sensing Systems in Food Safety Detection.

Category	Target Analyte	Material System	Analytical Performance	Key Innovation	Ref.
Pesticide residues	Atrazine	MIP/rGO/PPy electrochemical composite sensor	Rapid detection with excellent linearity and reproducibility	Biomimetic recognition by MIP combined with highly conductive hybrid structure for enhanced electron transport	[92]
	Broad-spectrum organophosphate pesticides	Flexible PLA substrate with screen-printed electrodes	Wearable, in situ, and nondestructive detection	Direct attachment to fruit and vegetable surfaces enabling on-site monitoring	[93]
	Methyl parathion	MIP-Bi_2_WO_6_ QDs/COF photoelectrochemical sensor	Detection limit down to femtomolar level	Heterojunction-assisted photogenerated electron amplification significantly enhances sensitivity	[94]
Antibiotic residues	Sulfathiazole (SFT), Sulfamethoxazole (SFM)	TA-Au-Ag-ANpM/f-MWCNTs/poly(L-serine) composite electrode	Detection limit at picomolar level; recovery 95–102%	Au-Ag alloy and CNT synergy enhances electrocatalytic activity	[95]
Heavy metal ions	Pb^2+^, Hg^2+^	Chitosan/aptamer molecularly imprinted polymer (MIP)	Highly selective detection; stable signal in pH 6–8	Aptamer conformational stabilization + MIP-assisted molecular recognition improves anti-interference performance	[96]
	Cd^2+^, Pb^2+^, Hg^2+^	MXene@CeFe-MOF-NH_2_ electrochemical sensor	Nanomolar detection limits; excellent signal stability	Enrichment-driven high surface area composite enables simultaneous multi-metal ion detection	[97]
Foodborne pathogens	Salmonella, E. coli O26:B6, O111:B4 lipopolysaccharides	pHEMA-based SERS sensor with machine learning	Detection limit 0.7 μg/mL; 100% classification accuracy	Non-specific polymeric capture coupled with spectral pattern recognition via machine learning	[98]
	Staphylococcus aureus	Dual-mode BIPs-PEC photoelectrochemical biosensor with machine learning	Detection limit 1.06 CFU/mL; excellent anti-interference performance	Active/passive dual-mode sensing with intelligent signal interpretation	[99]

**Table 4:** Applications of Polymer-Based Sensing Systems in Environmental Monitoring.

Target Analyte	Material System	Detection Principle/Mechanism	Performance Features and Analytes	Ref.
Comprehensive water quality (pollution warning)	Polypyrrole (PPy)-enhanced artificial electroactive biofilm (Shewanella oneidensis)	PPy improves conductivity and electron transfer efficiency, regulating electron-proton coupling in biofilms	Significantly enhanced sensitivity, enabling rapid water quality warning	[100]
Nitrogen pollution in water	Electrically stimulated anaerobic ammonium oxidation (anammox) biofilm	Weak electric field regulates biofilm structure and electron transfer pathways	Enhanced nitrogen removal rate and system stability	[101]
Heavy metals (Hg^2+^, Pb^2+^)	Polyaniline-Reactive Yellow 42 dye composite-modified electrode	Electron transfer enhancement verified by DFT calculations	Simultaneous detection of Hg^2+^ (LOD 2 nM) and Pb^2+^ (LOD 6.2 nM); applicable to complex water samples	[102]
Multi-parameter water analysis (BOD, toxicity)	Nanocomposite conducting polymer-microbial consortium system	Synergistic amplification of biocatalytic reactions and nanoscale conductive pathways	Simultaneous detection of BOD and toxicity with high sensitivity and rapid response	[103]
Organic pollutants (p-nonylphenol, PNP)	Laccase-immobilized conducting polymer electrode	Enzymatic oxidation-electron transfer coupling mechanism	Nanomolar detection limit; ≈100% recovery in real water samples; suitable for endocrine disruptor monitoring	[104]
Gaseous pollutants (CH_4_, CO_2_)	Dual-cell solid electrolyte electrochemical sensor	Gas-electrolyte interfacial reactions induce potential difference	Simultaneous CH_4_ and CO_2_ detection; response < 1.5 min; suitable for high-temperature industrial environments	[105]
Multiple gaseous pollutants (CO, N_2_O, O_3_, CH_4_)	Graphene-polyimide (Kapton) flexible gas sensor	Gas adsorption induces resonance frequency shift in graphene	High sensitivity, low power consumption (1.5 mW), and excellent flexibility for noninvasive environmental monitoring	[106]

## Data Availability

No new data were created or analyzed in this study.
